# Conference Report: WORKSHOP ON AGING, DIMENSIONAL STABILITY, AND DURABILITY ISSUES IN HIGH TECHNOLOGY POLYMERS Gaithersburg, MD May 28–29, 1992

**DOI:** 10.6028/jres.098.036

**Published:** 1993

**Authors:** Cynthia Arnold-McKenna, Gregory B. McKenna

**Affiliations:** White House Office of Science and Technology Policy, Old Executive Office Building, Room 427, Washington, DC 20500; National Institute of Standards and Technology, Gaithersburg, MD 20899-0001

## 1. Introduction

The expected functional life of polymers and polymer-based composites subjected to a set of environments and stress conditions is often deter- mined by issues of aging, dimensional stability and durability. The complexity of these issues leads not only to a need for further research developments but also improved methods of application of current physical and chemical knowledge to the design, manufacture, and use of materials. The knowledge base required to attain improvements in materials performance requires a “high technology” approach across the entire spectrum of industries that critically depend upon materials.

Problems associated with the lifetime and performance of polymers and polymer-based composites are manifest in many products and industries ranging from automotive and aerospace to photographic and imaging applications and electronic packaging. In some cases, the product design is necessarily conservative to compensate for the lack of understanding of the base materials. In other cases, the developments required to advance into new markets are constrained because testing methodologies are unavailable to benchmark material performance. Furthermore, materials design cannot advance in the absence of a fundamental understanding of the mechanical performance of materials.

Materials suppliers, end-users, and government experts were brought together at a workshop sponsored by the Materials Science and Engineering Laboratory of the National Institute of Standards and Technology, held at NIST on May 28-29, 1992. The purpose of the workshop was to provide inputs and advice into the programs at NIST involved in the study of the mechanical performance of polymers. The participants in the workshop began a dialogue on the possible mechanisms of interaction between industry and government, viz., NIST, to address these problems in a mutually beneficial way. The long term goal is to enhance the knowledge base associated with materials performance, with the result of improving the competitiveness of U.S. industries that critically depend upon materials performance.

In this conference report, we highlight the presentations made at the workshop in a format that first presents a scientific perspective on the issues, then highlights manifestations of the problems experienced across industries, indicating the ubiquitous nature of the relevant phenomena. Finally, we summarize the technological difficulties in the application of polymers and polymer-based composites that need to be addressed and surmounted in order to positively affect the industries involved.

## 2. The Science Base of Performance Issues

**Fig. 1:**
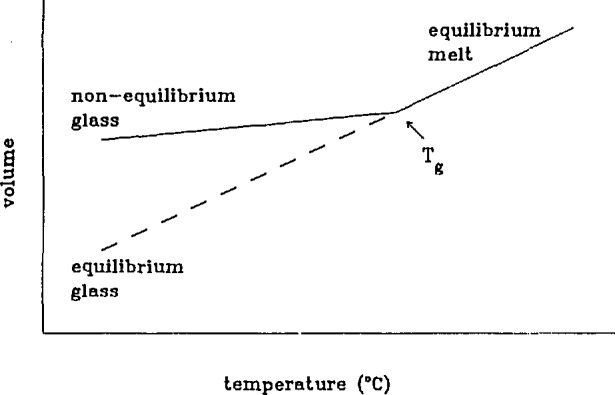
Schematic representation of volume—temperature behavior of a glass forming material.

The implications of the process of physical aging on long-term performance of polymeric glasses were outlined in an introductory presentation by Dr. G. McKenna (NIST), who highlighted the research on physical aging that has been an ongoing activity at NIST for about the past decade. In Fig. 1, the thermodynamic basis for physical aging is delineated. When a glass-forming polymer is quenched from above to below its glass transition (*T*_g_), it becomes essentially trapped in a non-equilibrium state [1,2]. (This is an event which occurs in the manufacture of nearly all polymers.) With time, the glass evolves towards equilibrium, and this volume recovery results in the process known as physical aging [3]. The importance of physical aging can be put into perspective in that it affects many, if not all properties of materials, such as dimensional stability, creep compliance, the relaxation modulus, yield strength, and fracture and failure resistance.

**Fig. 2:**
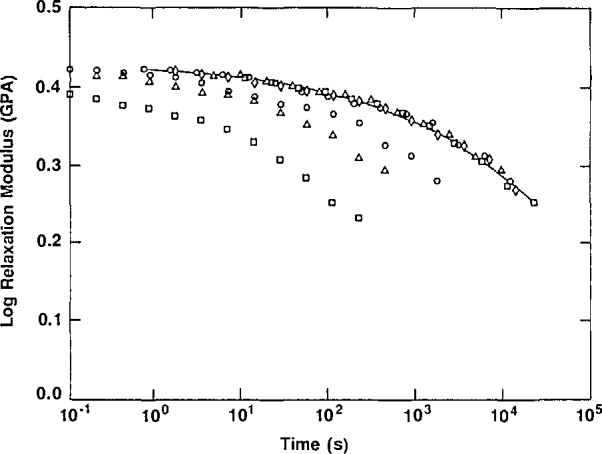
Relaxation modulus vs time at different aging times for an epoxy glass quenched from above the glass transition temperature to 25 °C below it. Aging times after the quench are (□) 28 min; (Δ) 125 min; (○) 503 min; (◊) 4026 min. Solid line depicts curve obtained from time-aging time-superposition. (After Ref. [4])

**Fig. 3:**
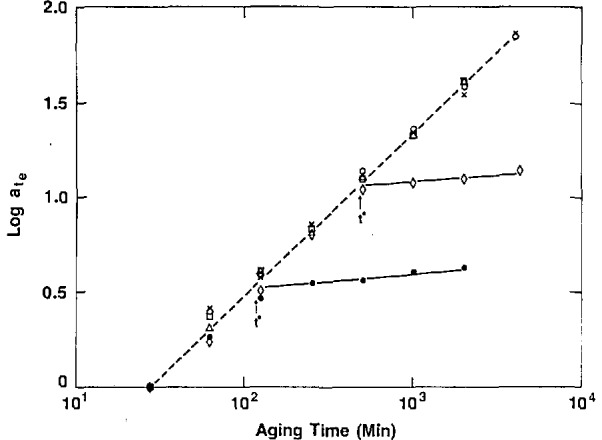
Double logarithmic representation of aging time shift factor (*a_t,e_*) vs aging time (*t*_e_) for an epoxy glass quenched from above *T*_g_ to different temperatures below *T*_g_. T: (●) 66 °C; (◊) 62 °C; (Δ) 57 °C; (○) 42 °C. Note that near to *T*_g_ = 72°C the aging virtually ceases at t*. (After Ref. [4])

McKenna discussed the work carried out at NIST on aging of epoxy resins in the context of the classical [3] time-aging time superposition concept. Thus, as depicted in Fig. 2, the relaxation modulus for an amine-cured epoxy evolves with aging time, *t*_e_, after a quench. All of the curves can be overlaid to form a single “master curve” by shifting them along the horizontal axis. The amount of displacement required for each curve to overlay on the master curve is called the “shift factor,” denoted *a*_te_. The ability to shift each curve is referred to as time-aging time superposition and is important in developing mathematical models of the process. In Fig. 3, the “shift factor” is plotted as a function of the aging time. Importantly, near the glass transition, aging eventually stops as the glass attains equilibrium. However, far below *T*_g_, aging continues for extremely long times *at the same rate –* a factor that is important in long term applications of polymers.

Although the linear response of polymeric glasses to physical aging is readily understood within the context of time-aging time superposition principles, the same cannot be said of the nonlinear response that is relevant to material behavior in fatigue and fracture (large deformations at crack tips), residual stresses in composites, and yield of engineering resins. The current controversy has strong implications for the development of nonlinear constitutive equations for polymeric materials and the physical basis for the mathematical functions upon which these are based.

According to L. C. E. Struik [3], large stresses and deformations “erase” the prior aging or “rejuvenate” the glass. Struik argued that the large mechanical stimuli cause the thermodynamic state of the glass to be changed to one that looks more freshly quenched. The evidence for this was that the slope of the logarithmic plots of *a*_te_ vs *t*_e_ (see Fig. 3) was reduced when the aging was probed using large stresses.

**Fig. 4:**
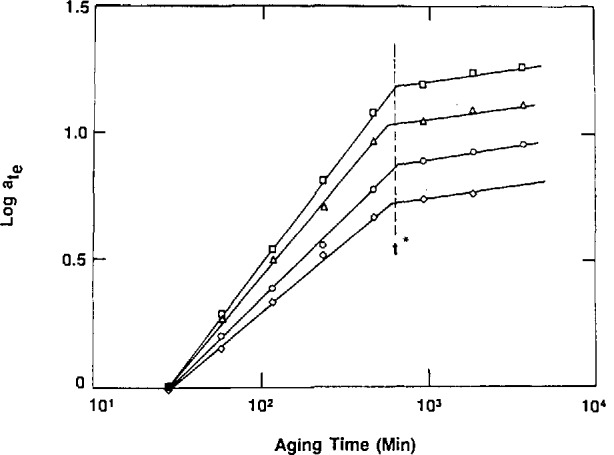
Double logarithmic representation of aging time shift factor (*a*_t,e_) vs aging time (*t*_e_) for an epoxy glass quenched to 5 °C below its glass transition and probed by different magnitude stresses. (□) 1 MPa; (Δ) 5 MPa; (○) 10 MPa; (◊) 15 MPa. Note that t* does not vary with the magnitude of the applied stress, although the slopes of the data prior to t* do. (After Ref. [5])

McKenna described two types of experiment performed at NIST that contradict Struik’s hypothesis. First, by working close to the glass transition, the kinetics of aging could be characterized not only by the value of *μ* = d log *a*_te_/d log *t*_e_, as had Struik, but also by the value of t*, the time required for the viscoelastic properties to cease evolving. As shown in Fig. 4, t* is independent of the applied stress [5], whereas the Struik hypothesis [3] would have demanded that t* increase as stress increases.

In the second set of experiments a torsional dilatometer was used to simultaneously measure the volumetric and mechanical response subsequent to a quench from above to below *T*_g_. Again contradictory to Struik [3], the application of the mechanical deformation did not affect the time evolution of the volume of the glass towards equilibrium. Rather, the volume recovery which causes aging affects the nonlinear response (large stress) of the glass less than it does the linear response (small stress).

The implications for the above evidences are that the glassy structure has a large effect on the mechanical response, in particular, in the small deformation regime. The effects of glassy structure on the non-linear response are less important. However, large mechanical stimuli do not alter the underlying structure of the glass. This is an important conclusion because it simplifies greatly the description of the viscoelastic response of glasses and the constitutive equations needed to model the properties of materials.

**Fig. 5:**
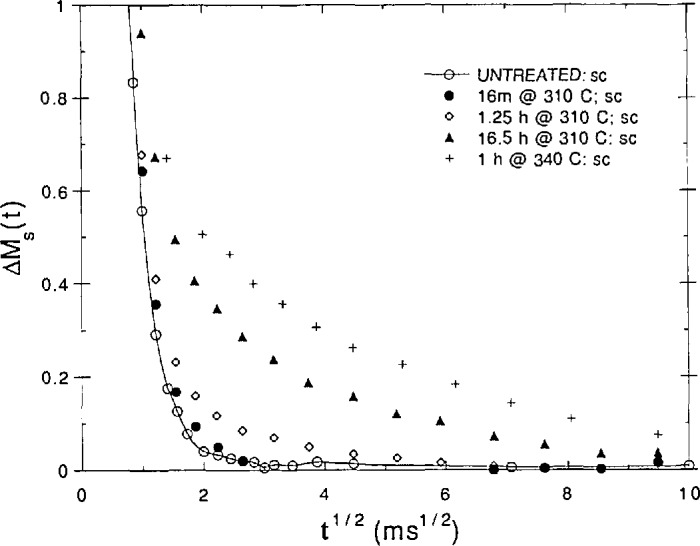
NMR spin diffusion results for a PEI/PBI blend that was initially homogeneous (untreated) showing effects of aging time and temperature on phase separation. (See text for discussion). (D. L. VanderHart, NIST.)

Dr. D. VanderHart (NIST) discussed the use of nuclear magnetic resonance (NMR) spectroscopy to characterize structural features and the kinetics of their formation in glassy polymer blends. NMR offers advantages over small angle x-ray scattering, which is ineffective if the electron densities of the phases are similar. It can also provide chemical information about the individual phases. Spin diffusion NMR was used to obtain structural information, e.g., phase size and stoichiometry. In a study of the high temperature behavior of poly(etherimide)–polybenzimidazole (PEI/PBI) blends, VanderHart showed the importance of the kinetics of the glass transition phenomenon on the long term phase stability of these blends. In Fig. 5 is plotted the spin diffusion results for different thermal treatments as Δ*M*_s_ (the difference in the magnetization from the equilibrium magnetization) vs time for the PEI phase aliphatic protons. The time for Δ*M*_s_ to return to zero provides an estimate of the domain size. The untreated, homogeneous system shows a very rapid decay to zero as the spins equilibrate rapidly due to the intimate mixing of the PEI and PBI chains. As the aging time gets longer or the temperature higher, the decay time gets longer because spin equilibration occurs due to diffusion from the PEI phase to the PBI phase, consistent with the phase separation of the system. This occurs even though these experiments were performed well below *T*_g_ for the blend of 347 °C. Such results have serious implications for the long term performance of materials because the fundamentally kinetic nature of the glass transition implies that short term results are not readily extrapolated to long times. Thus, blends can apparently phase separate at long times well below *T*_g_.

Clearly, much fundamental information is known about the time-dependent properties of polymers. Incorporating this knowledge into adequate descriptive models by the engineering design community has not kept pace. Therefore, predictive models for the long-term in-service performance of polymers and polymer-based composites that are needed by materials suppliers and end-users are lacking. The science base also needs to be advanced. Chemical and physical mechanistic changes which are associated with a loss of performance and failure, such as plasticization, recrystallization, degradation of molecular weight, and stress cracking, affect the bulk state. These complex relationships must be defined, as failure is not obviously related to singular changes in the bulk state. The models must deal with the additional complexities of heterogeneous structures that are process dependent; the synergistic effects of aging, load, temperature, and solvents; and the added dimension of time scales.

## 3. Approaches to Addressing Performance Problems

While the scientific understanding of some aspects of aging, dimensional stability, and durability of polymers and polymer-based composites is well in hand, there are other areas in which such is not the case. The range of approaches to these issues represented by the workshop speakers demonstrates this. In some instances, the complexity of the issues (in particular, synergism of effects of nonlinearity of the phenomena) makes even parametric approaches appear virtually incomprehensible. In other instances, the application of fundamental understanding to specific problems can help solve them, post-facto. Overall, the trend that becomes apparent is that not only is there a need for more fundamental science, but there is a need for better application of the fundamental science early in the design process to avoid later stage problems in the manufacturing and service stages of material lifetimes.

### 3.1 Identification of General Needs

**Fig. 6:**
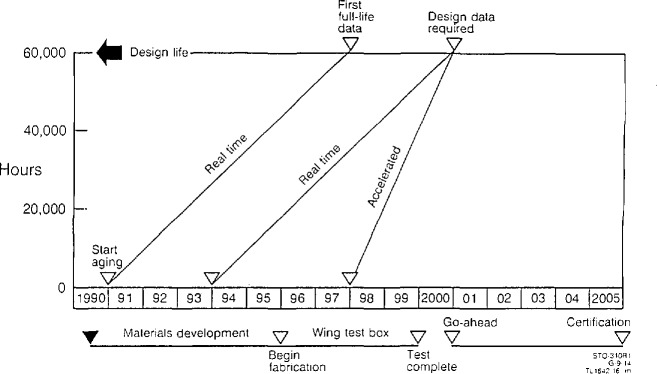
Representation of the schedules for development of HSCT and how real-time materials testing and accelerated testing methodologies (currently non-existent) affect HSCT go-ahead for manufacturing commitment. (D. Grande, Boeing Company.)

Some key technical issues in bringing new polymers and polymer-based composites into market applications were examined by representatives from a materials end-user, a materials supplier and a government agency. Mr. D. Grande from Boeing Company discussed the implications of aging and durability for meeting the materials requirements for composite structural applications in the high speed civil transport (HSCT). Mr. Grande identified the key technical barriers to the commercialization of this second generation supersonic transport as the understanding of aging effects in polymer-based composites and the need for low cost design and manufacture of composite structures. The demanding service environment of the aircraft requires durability for 12,000 supersonic hours under cyclic loads and temperatures up to 175 °C. Ideally, under these conditions, material properties will not change. However, noting the time-dependent nature of polymer properties, particularly in aggressive mechanical, thermal and chemical environments, the prediction of lifetime performance becomes mandatory. The aging issue is shown schematically in Fig. 6. For materials development to be successful, the seven year lead required for real-time testing of components must give way to successful accelerated testing methodologies and predictive models. Thus, for an HSCT to be given a “go-ahead” in the year 2000, real time testing of current (1993) materials must already be underway. Accelerated testing would allow the introduction of improved materials as late as 1997. As current materials are already known to be inadequate to meet the demanding performance requirements of the HSCT, the lack of models for long-term performance of polymer-based composites will delay the commercialization of the aircraft. Data in the absence of fundamental knowledge leads to a trial and error development cycle that is prohibitively costly. The heterogenous nature of composite systems and synergies of the multivariate environmental exposures further complicate the development of coherent test methodologies and predictive models for composite performance. Finally, a fundamental understanding of the manufacturing processes has not been established.

The problems which these circumstances present to a materials supplier were highlighted by Dr. C. Carlson (DuPont). The lack of understanding of the aging process and its effects on materials properties result in serious economic ramifications. The development of high technology polymers must be science-based and include the analytical tools and methods, and modeling and simulations capabilities, to avoid, as Mr. Grande suggested, the trial and error materials development which otherwise results. A knowledge of the applications and global market needs also contribute to successful, timely development of materials. However, the development cycle time must be minimized, as it is difficult to attract and sustain resources throughout a long development-to-commercialization cycle. For example, DuPont’s Avimid K-3 polyimide series, first developed in 1982, has not been utilized in production; opportunities for large-scale commercialization will be available no sooner than 1997—at least 15 years after the initial development. Currently, product validation requires real-time testing. Accelerated testing methodologies and predictive models for materials performance need to be developed for pure materials, composites, and structures.

While the modeling needs and lack of adequate accelerated testing methodologies in advanced materials are obvious in the above, the same problems also occur in lower performance polymers and their composites in other industries. The high technology needs in automotive applications were outlined by Dr. J. Eberhardt of the U.S. Department of Energy. He noted that many materials properties change with time due to the mechanical, environmental, and thermal service environment, which is delineated in Table 1 for various automotive applications. Many modes of damage are experienced: impact, fatigue, temperature-induced creep, delamination, chemical attack, and importantly, synergism among the modes. Coupon tests provide data on singular effects, but synergistic effects are not duplicated with coupons. Adhesive durability is also an issue. Recognizing that the average age of the American automobile has increased almost 40% since 1970, Dr. Eberhardt expressed that the major needs for understanding lifetime performance issues in these applications are realistic accelerated and application-specific test methods. His concerns and interests were reiterated by the industrial representatives to the workshop.

### 3.2 Semi-Empirical Approaches

The important need for test methodologies and predictive models was highlighted in several presentations in which semi-empirical approaches were described which addressed immediate problems in design, application and manufacturing. Currently, prediction of long-term performance of materials is frequently done by straight-line extrapolation of short-term data, gathered from tests which simulate, as closely as possible, the actual in-service environment. For example, Dr. D. Houston from Ford Motor Company indicated that test coupons of materials for automotive applications are currently tested by mounting them on undersides of trucks in various cities in the United States with widely different climates, such as Phoenix and Detroit, and driving them for various times. After such in-service exposure, the physical/mechanical properties are determined and incorporated into the design criteria.

**Fig. 7:**
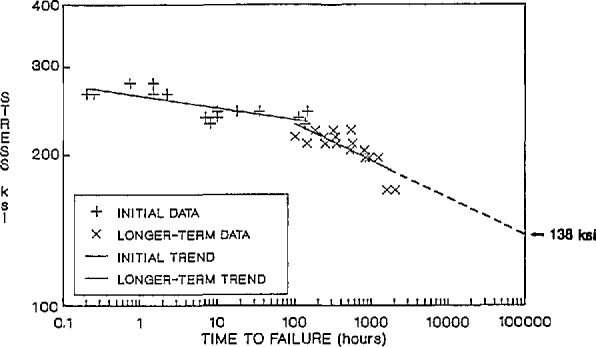
Stress at rupture vs time to failure for a composite rod showing how short time data (< 100 h) could not be extrapolated to predict long term performance. (M. Greenwood, Owens-Corning.)

Without insight into the physical and chemical processes that are occurring, some of which are nonlinear, semi-empirical approaches can lead to erroneous predictions of performance. For instance, Mr. M. Greenwood (Owens-Corning Fiber-glass) discussed the need and approaches of the civil engineering community for accelerated tests and failure prediction. In Fig. 7, an apparent change in mechanism(s) influences the stress at rupture for a composite rod exposed to an aggressive environment during testing. The degradation rate increases dramatically at times longer than 100 h. Thus, long term performance could not be predicted by a simple (straight-line) extrapolation of the short term data. For improved prediction of durability, other considerations, such as sample preconditioning prior to testing, should be included in the methodology.

The discussion by Drs. N. Kakarala (General Motors), Houston and Greenwood indicated that tests are frequently performed in more severe environments than the average service environment in order to simulate long-term exposure in short times. Without insight into degradative mechanisms, the development of such tests is severely flawed. Furthermore, statistical uncertainty in the measurements leads to overly conservative designs. Some desirable criteria for developing test methodologies were delineated: to reflect the service conditions; for samples to be representative of the product with regards to the materials and manufacturing “quality”; and test and service failure modes should be the same.

Different aspects of the complexity of predicting the performance of polymeric materials were illustrated in the work on electronic packaging materials discussed by Dr. C. Lee of the MCC Packaging and Interconnect Program. He tabulated a variety of property data as a function of processing (cure) conditions. Property measurements included residual stress, the glass transition, and the coefficient of thermal expansion (CTE). These were affected by the curing temperature and rate, and the annealing temperature. The limitations of such a semi-empirical approach can be seen from the following example given by Dr. Lee. In one instance, the CTE of packaging material was found to have a minimum when aged near *T_g_*, while another system was found to either have an increase or a decrease upon aging, depending on the exact nature of the curing process. Such results are confusing when the fundamental relationships between the properties, in this case CTE and the process history, have not been adequately developed. In particular, complex interactions between the chemistry of curing systems and the aging process need to be considered in detail if one is to successfully predict properties that are relevant to device performance.

The specific effect of physical aging on the creep of the engineering polymers Noryl—a blend of poly(phenylene oxide) and high impact polystyrene — and polycarbonate was studied by Dr. G. Tryson of General Electric. Given that creep is moderated by physical aging, Tryson noted that a product may be overdesigned if physical aging is not considered in the design process. In attempting to develop a meaningful correlation between aging and creep performance, he noted some variables that were difficult to evaluate; fillers, additives, fibers, and other modifiers; non-constant loads and temperatures; residual stress of the component; and the effects of aging on the creep rupture performance.

### 3.3 Science-Based Approaches to Problem Solving

As discussed in Sec. 2, there is a considerable, albeit incomplete, science base to our understanding of the problems of aging and dimensional stability of polymeric materials. As a result, several of the workshop speakers were able to present approaches used in their companies that took into account the science of aging to improve manufacturing or to resolve specific problems once they had arisen.

Dr. G. Pearson of the Eastman Kodak Company gave an extensive overview of the importance of aging in both cellulose triacetate and poly(ethylene terephthalate) in photographic films.

**Fig. 8:**
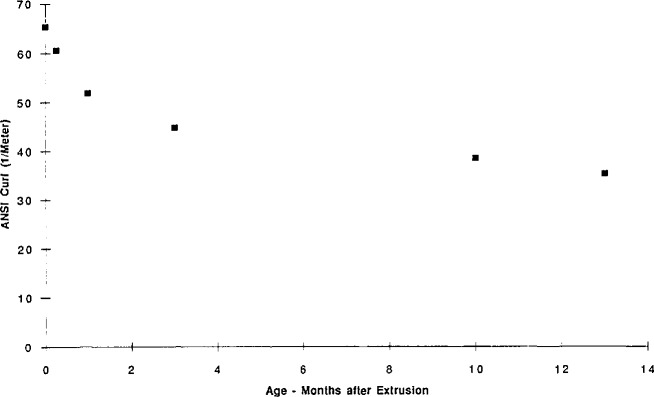
ANSI curl number vs aging time for extruded poly(ethylene terephthalate). (G. Pearson, Eastman Kodak Company.)

In addition to showing that aging affects a variety of engineering properties, such as the ANSI curl number (a measure of the viscoelastic set of a film) as shown in Fig. 8, Pearson also described how learning to control aging became important for the manufacturing of films as Kodak moved towards tighter inventory control and just-in-time inventory/delivery procedures. The shortening of the holding time between manufacture and actual delivery of films makes control of the film properties and dimensions more important. Where previously films sat in warehouses long enough to age or stabilize, the rapid turnover could lead to “unaged” or “partially aged” films being delivered. The difference in aging could result in a variable product. Having developed a fundamental understanding of the relationships between aging and the properties of interest, the manufacturing parameters could be modified to assure that high quality products were consistently delivered.

Some of the problems involved in epoxy-novolac encapsulants for integrated circuit devices were described by Dr. H. Bair of AT&T Bell Laboratories. He discussed the importance of keeping moisture away from the resins during cure. High moisture content resulted in large decreases in *T_g_* and the extent of cure. In addition, upon exposure to elevated temperatures, greater permanent changes in encapsulant dimensions (expansion) occurred when the encapsulant was cured in the presence of moisture than when dry. By processing in the absence of moisture, the final properties of the system could be controlled and the quality of the products guaranteed. Improvements in dimensional and property stability could be obtained by using encapsulants with higher glass transitions or by using resins where cure chemistry was less moisture-sensitive.

**Fig. 9:**
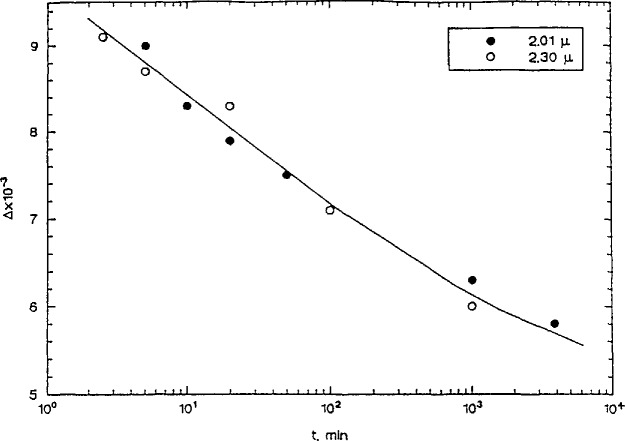
Birefringence of an oriented polycarbonate material as a function of time after processing at 411 K. (W. Prest, Xerox Corporation.)

Dr. W. Prest of the Xerox Corporation discussed the effects of physical aging on the optical, electrical, and mechanical properties of imaging components. The densification that accompanies aging increases the polarizability per unit volume, producing corresponding changes in the index of refraction of optical components. Fig. 9 illustrates the change in the birefringence as a function of aging time. However, the anisotropy stops changing before the index of refraction. The densification also increases charge mobility, as the inter-site hopping distance between dispersed electrically active species is decreased. The accompanying aging induced restriction in molecular mobility (aging rate) gives rise to changes in the yield and fracture behavior of the polymeric glasses, affecting durability and the modes of failure; brittle fracture is associated with long aging time, for instance.

### 3.4 Science-Based Predictive Approaches

**Fig. 10:**
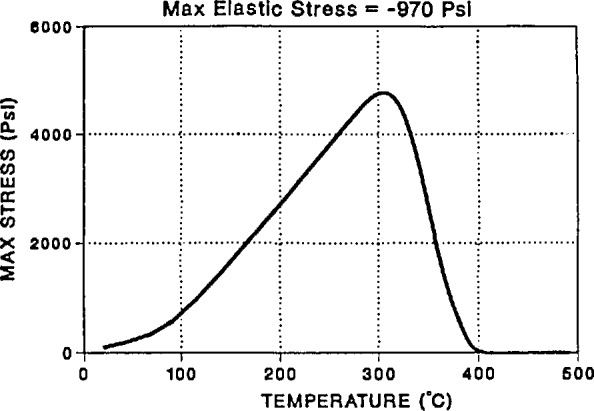
Stress in the glass in a glass/metal seal upon cooling as calculated using a finite element analysis incorporating volume recovery of the glass. Note that the stresses go through a tensile maximum at approximately 300 °C. Linear thermo-elasticity predicted a compressive stress throughout the thermal cycle. (R, Chambers, Sandia National Laboratory.)

Dr. R. Chambers from Sandia National Laboratories in Albuquerque presented results from finite element analyses that he has performed on model glass/metal seals. The goal here is to design systems in which the glass, after processing at a high temperature to form the seal, is in a state of compression at low temperatures. Residual tensile stresses can lead to failure of the seal. Chambers applied the Narayanaswamy [7] nonlinear model of volume recovery of glass-forming materials in his finite element analysis and compared it to simple linear models of thermo-viscoelasticity. Chambers showed that the nonlinear modeling indicates a tensile stress in the glass during cooling while the linear analysis predicted compressive stresses. (See Fig. 10.) This correlated well with the observation of cracking due to tensile stresses during the processing of real glass to metal seals that had been designed on the basis of a linear thermoelastic model.

The importance of the Chambers modeling is that it takes the current knowledge of physical aging of glass-forming materials (Narayanaswamy’s volume recovery model) and applies it to a composite system (glass/metal seal) in the framework of the design engineer (finite element analysis). Although the model itself was simple, it points the direction to be taken for more complicated fiber-reinforced composites. Chambers also noted the need for materials property data for volume recovery of materials used in composites, as well as better constitutive models to describe the non-linear mechanical response of glass-forming materials. Further experimental work is needed to validate finite element models of more complex systems.

The science base of durability issues was addressed by Dr. C. Bosnyak (of the Dow Chemical Company). Reiterating the needs identified by the preceding speakers, Bosnyak noted that all models of durability require as their basis the fundamental principles and mechanisms of the relevant physical and chemical phenomena, adding that the prediction of durability has not been developed as a technology. For models to be useful, they must be *predictive*, rather than solely descriptive; however, at least one participant remarked that even adequate descriptive models are currently unavailable. Bosnyak highlighted — in agreement with the participants—that models that are derived from databasing and curve-fitting have limited utility to guide the work of materials suppliers, designers, and users. Suppliers have the ability to challenge the utility of models by applying their extensive materials databases to test them; users may also bound the problem by defining the failure criteria, based on the material’s application.

Bosnyak identified the scientific criteria necessary for developing lifetime prediction models, starting with an intimate knowledge of the application and materials. The variables that need to be understood are outlined in Table 2. Emphasis was placed on understanding and quantifying the mechanisms of failure, and relating these to the structure of the material. The next step links the results of laboratory tests to performance, yielding the ability to predict in-service performance. This could be accomplished by developing scaling concepts of time and geometry, from which accelerated test methodologies could be used to simulate long-term performance and failure.

**Fig. 11:**
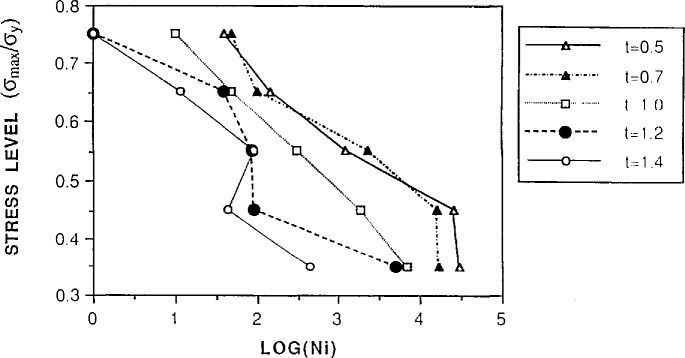
Stress-crack initiation-cycles diagram for polycarbonate showing how different failure mechanisms intervene depending on the geometry of the specimen. Thickness of specimens is indicated beside diagram. (The crack initiation time (Ni) was defined as the time when the crack length was 0.2 mm on the specimen surface from the V-notch tip under optical microscope observation.) (C. Bosnyak, Dow Chemical Company.)

**Fig. 12:**
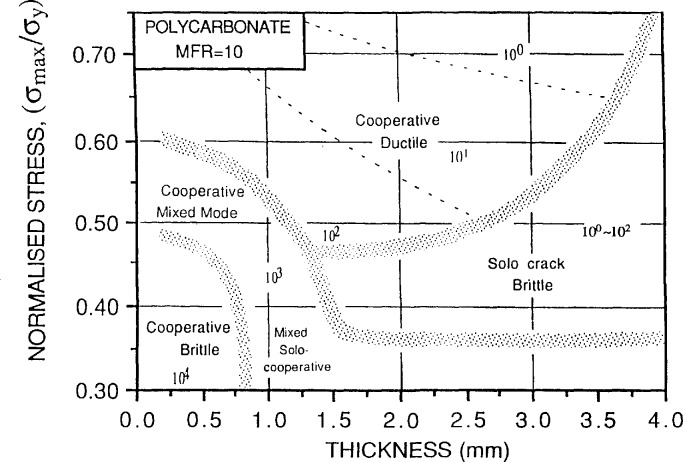
Fatigue crack initiation mechanism map for polycarbonate showing how failure mode depends on both level of applied stress and the specimen thickness. (C. Bosnyak, Dow Chemical Company.)

In his research, Bosnyak examined the role of failure mechanisms in the development of accelerated test methodologies for polycarbonate. He developed a diagram of the stress vs crack initiation time for samples of varying thickness (Fig. 11). As noted by previous speakers, simple data extrapolation would lead to erroneous conclusions, in this case, the crack initiation time. However, by normalizing the stress and “mapping” regions in which different failure mechanisms were exhibited, a useful fatigue crack initiation mechanism map was developed (Fig. 12). Thus, for known geometries, loads, and mechanisms of failure, the crack initiation time is predictable. However, although the mechanism maps represent a significant advance toward understanding durability in polymers, it is only the first step. Further work is ongoing to select appropriate normalization of the axes. Importantly, it should be clearly recognized that durability or toughness cannot be adequately described by a single number.

## 4. Summary and Conclusions

An industry/NIST workshop brought together industrial and government scientists, engineers, and technical managers to assess the state and application of knowledge in addressing a range of problems associated with aging, dimensional stability, and durability of high-technology polymers. The workshop participants, representing 16 companies, as well as NIST and the Department of Energy, compared experiences and perspectives on a class of problems that are ubiquitous in polymer science and technology and impact a range of products and processes in many industries, from photographic and imaging applications to electronic packaging and composites for automotive and supersonic commercial aviation applications. A process, termed physical aging, in which the dimensions and performance properties of polymers change with time in service, was considered a critical scientific problem that should be addressed in collaborative work between industry and NIST.

The workshop participants noted that although much fundamental information is known about the time-dependent properties of polymers, this knowledge has not, for the most part, been incorporated into adequate descriptive models by the engineering design community. Furthermore, models must be predictive, rather than merely descriptive, in order to be useful to materials suppliers, designers, and users. The development of predictive models is even less advanced, as evidenced by a common approach to the prediction of long-term in-service performance described by several workshop speakers: the straight-line extrapolation of short-term data gathered from tests which simulate, as closely as possible, or intensify the actual in-service environment. This approach frequently fails due to a lack of fundamental physical and chemical mechanistic information, particularly concerning non-linear behavior. The utility of this approach is further limited by the complexities of structural heterogeneities that are process-dependent; the synergistic effects of aging, load, temperature, and solvents; and the added dimension of time scales. Thus, the basic science associated with performance issues must be advanced; the workshop participants suggested that NIST could play a valuable role by addressing these issues to support the development of predictive models and accelerated test methodologies.

## Figures and Tables

**Table 1:** Applications and operating environments for polymers and composites in automotive applications (J. Eberhardt, DOE)

Operating environment	Mechanical	Environmental	Thermal
Materials application			
Interior materials	Vibration	UV and visible radiation	Solar heat load
Exterior materials	Impact loads Vibration Structural weight	UV radiation Ozone Chemicals	Outside temp. Direct sun
Underhood and under chassis	Cyclic loads Impact loads Vibration	Chemicals Ozone	Hot engine Solar heat load

**Table 2:** Key variables in the engineering design for plastics (C. Bosnyak, Dow Chemical Company)

Material	Geometry	Loading	Environment
Molecular wt (MW)	Thickness	Stress level	Temperature
MW distribution	Shape (stress concentration)	Stress rate	Weathering
Morphology		Rate	Chemicals

## References

[1] Kovacs AJ (1963). Fortsch Hochpolym Forsch.

[2] McKenna GB, Booth C, Prize C (1989). Comprehensive Polymer Science. Polymer Properties.

[3] Struik LCE (1978). Physical Aging in Amorphous Polymers and Other Materials.

[4] Lee A, McKenna GB (1988). Polymer.

[5] Lee A, McKenna GB (1990). Polymer.

[6] VanderHart DL, Campbell GC, Briber RM (1992). Macromolecules.

[7] Narayanaswamy OS (1974). J Am Ceram Soc.

[8] Chambers RS, Gerstle FP, Monroe SL (1989). J Am Ceram Soc.

